# Developing a CNT-SPE Sensing Platform Based on Green Synthesized AuNPs, Using *Sargassum* sp.

**DOI:** 10.3390/s20216108

**Published:** 2020-10-27

**Authors:** Fanny J. González-Fuentes, Gustavo A. Molina, Rodolfo Silva, José Luis López-Miranda, Rodrigo Esparza, Angel R. Hernandez-Martinez, Miriam Estevez

**Affiliations:** 1 Centro de Física Aplicada y Tecnología Avanzada, Universidad Nacional Autónoma de México, Boulevard Juriquilla 3001, Querétaro, Querétaro 76230, Mexico; fanny.gonzalez@fata.unam.mx (F.J.G.-F.); lopezfim@fata.unam.mx (J.L.L.-M.); resparza@fata.unam.mx (R.E.); angel.ramon.hernandez@gmail.com (A.R.H.-M.); 2 Posgrado en Ciencia e Ingeniería de Materiales, Centro de Física Aplicada y Tecnología Avanzada, Universidad Nacional Autónoma de México, Boulevard Juriquilla 3001, Querétaro, Querétaro 76230, Mexico; gamol@fata.unam.mx; 3 Instituto de Ingeniería, Universidad Nacional Autónoma de México, Edificio 17, Ciudad Universitaria, Coyoacán 04510, Mexico; rsilvac@iingen.unam.mx

**Keywords:** carbon nanotubes-screen printed electrodes, electrochemistry, *Sargassum* sp., green synthesis, nanoparticles, sensing platform

## Abstract

Detection and quantification of diverse analytes such as molecules, cells receptor and even particles and nanoparticles, play an important role in biomedical research, particularly in electrochemical sensing platform technologies. In this study, gold nanoparticles (AuNPs) prepared by green synthesis from *Sargassum* sp. were characterized using ultraviolet-visible (UV-Vis) and Fourier transform-infrared (FT-IR) spectroscopies, X-ray diffraction (XRD), scanning electron microscopy (SEM), dynamic light scattering (DLS) and zeta potential (ζ) obtaining organic capped face-centered cubic 80–100 nm AuNPs with an excellent stability in a wide range of pH. The AuNPs were used to modify a carbon nanotubes-screen printed electrode (CNT-SPE), through the drop-casting method, to assemble a novel portable electrochemical sensing platform for glucose, using a novel combination of components, which together have not been employed. The ability to sense and measure glucose was demonstrated, and its electrochemical fundamentals was studied using cyclic voltammetry (CV). The limits of detection (LOD) and quantification (LOQ) to glucose were 50 μM and 98 μM, respectively, and these were compared to those of other sensing platforms.

## 1. Introduction

Ultrafine materials, where at least one of dimension is less than 100 nm, are known as nanoparticles (NPs) [1]. Their importance lies in the fact that the nano-dimension can influence the physicochemical properties of the material due to the high surface to volume ratio. NPs therefore have unique physicochemical properties, such as color, solubility, strength, diffusivity, toxicity, magnetic, optical, thermodynamic, catalytic, etc. For example, NPs of gold (Au), platinum (Pt), silver (Ag), and palladium (Pd) around 20 nm can present wine red, yellowish-grey, black and dark brown colors, respectively, but these same NPs present different colors at 80 nm [2].

Among metals, gold nanoparticles (AuNPs) have attracted considerable attention, and they have been widely used in biomedical applications due to their intrinsic features such as optical, electronic, physicochemical and surface plasmon resonance (SPR). Due to their ease of synthesis and functionalization properties, AuNPs have been employed in various applications in a range of biomedicine fields, such as sensing, targeted drug delivery, imaging, photothermal, and photodynamic therapy [3]. In chemical and/or biological sensing applications, AuNPs have been used for various types of platforms, especially in electrochemical sensors and biosensors [4]. These sensors are useful tools, which can detect low concentrations of different compounds in small sample volumes. They are highly selective and sensitive, and it can be easily miniaturized for the development of the portable analytical devices [5].

Electrochemical sensing platforms are useful for on-site, on-line, in-line, or real-time analysis. Their analytical performance characteristics are improved by using different electroactive compounds, nanomaterials (e.g., AuNPs), and/or enzymes to increase its selectivity and sensitivity. Much of the knowledge we have regarding the manipulation of nanostructured materials structure or composition and its relationship with the performance of the electrochemical sensors has focused on the study of carbon nanotubes, graphite, and AuNPs [6]. The study into manipulating nanoparticle structure or composition on AuNPs and its application in the development of novel sensing platforms has not been exhausted.

The increasing use of nanostructured materials sensing applications and technological applications has prompted concern about contamination in nanomaterial preparation, handling, and application processes, as well as in its release into the environment. To address this concern, various research groups have pursued different approaches: assessment of the nanomaterials in the environment through the study of their mobility, reactivity, ecotoxicity, and persistency [7,8,9], preparation of environmentally benign sustainable nanostructured materials through green chemistry. The crude extracts of plants contain secondary metabolites mainly, such as flavonoids, phenolic acid, alkaloids, and terpenoids, which are responsible for the bio-reduction of metal ions and synthesis of metallic nanoparticles [10,11,12].

In recent years there has been great interest in the use of *Sargassum* sp. from the Mexican Caribbean coast, because since mid-2014 there has been an abnormal influx of it, year after year, causing problems related to health, ecosystems, and socioeconomic issues [13,14]. The bioactive components derived from seaweeds, such as polysaccharides, pyropheophytin, tripeptides, phlorotannin, dioxynodehydroeckol, astaxanthin, flavonoids, terpenoids, phenols, and fucoxanthin are shown to have significant antioxidant properties, necessary for the synthesis of metallic nanoparticles [15,16].

Various nanostructured materials have been prepared using the green synthesis of metal nanoparticles (MNPs) using plant-based materials and substances [17,18,19,20]. These green-synthesized MNPs possess unique, self-assembling properties, greater surface to volume ratio, a size-dependent crystalline structure (1–100 nm), higher stability, specificity, encapsulation, surface structure, and biocompatibility due to their material structure MNPs/organic capping [21] when compared to chemically synthetized MNPs. Among the MNPs, AuNPs possess interesting optical and electronic properties, which give them high chemical stability and surface area, and which mean they could be used in bio-medic, catalytic, photonics, and sensing applications [19,22,23].

However, the MNPs/organic-capping structure, which is responsible for the excellent physicochemical and optical properties of the nanostructured materials, that we have previously reported, may also be responsible for a breakdown in charge transport between the AuNPs and the possible analyte in electrochemical sensing applications. The organic capping in the materials comes from the biomolecules extracted from plants and them with those responsible for carrying out the chemical processes of reduction of the metallic salts used as precursors. The chemical structure of this organic capping may contain areas with nucleophilic or electrophilic groups, which can improve or interrupt the charge transport process in electrochemical sensors and biosensors.

Glucose is an analyte of great interest. A monosaccharide present in the human blood, it has a very important role in metabolic processes, however an increase in its concentration can produce diseases [24,25]. According to the World Health Organization (WHO), the modern food industry should include information on the glucose content in food, for greater awareness and consumer expectations [26].

There are several techniques for glucose detection and quantification [27,28,29,30,31]. The effectiveness and disadvantages of these techniques depends on the enzyme immobilization method, which must assure stabilities, low cost and enzyme selectivity; long analysis times; employing large solvent volumes, high costs, and complexity in the treatment of results [32,33,34]. Consequently, it is important to develop fast, efficient, economical, and portable alternatives [35].

To address these issues, in the present project, we carried out the green synthesis of AuNPs using a *Sargassum* sp. (Ssp) aqueous extract as a reducing and stabilizing agent. Then, the obtained AuNPs have been deposited without removing the organic capping on the surface of carbon nanotubes-screen printed electrodes (CNT-SPE) for assembling a miniaturized and portable electrochemical sensor platform to evaluate their performance in detecting glucose.

## 2. Materials and Methods

### 2.1. Algae

Two species, *natans* and *fluitans*, make up the *Sargassum* sp. (Ssp), collected from the Mexican Caribbean coast. The sargassum was washed several times with distilled water, to remove any litter, impurities, and sand. Subsequently, it was dried in the shade for two weeks, then collected and placed into dry bags and sealed, then stored until required.

### 2.2. Chemicals

Gold (III) chloride analytical grade (HAuCl_4_*3H_2_O, >99.995% metal basis) was the precursor salt used, bought from Sigma-Aldrich. For the Sorensen Buffer preparation stock solutions sodium dihydrogen phosphate (NaH_2_PO_4_, ACS reagent, <99%) and sodium phosphate (Na_2_HPO_4_, ACS reagent) 0.2 M, were mixed to obtain a solution of pH 7. The reactants were bought from Macron Fine Chemicals^TM^. For the electrochemical determination, dextrose (D(+)-glucose, ≥99.5%), from Sigma-Aldrich, was used. For interference analysis of the platform, fruit sugar (D(−)-fructose, ≥99%) and citric acid (ACS reagent, ≥99.5%) were bought from Sigma-Aldrich. All the extracts, solutions, and dilutions were prepared with deionized water.

### 2.3. Preparation of the Sargassum sp. Extract

The Ssp extract was prepared using the infusion method; 2 g of the algae was placed in a beaker and 50 mL of distilled water was added. The mixture was mixed by magnetic stirring at 80 °C for 90 min. It was then cooled at room temperature. Whatman # 41 filter paper was used to separate the liquid phase and the extract obtained was stored at 4 °C in an amber glass vial for later use.

### 2.4. Green Synthesis of Gold Nanoparticles (AuNPs)

Next, 1 mL of precursor solution (HAuCl_4_) was mixed with the Ssp extract for the green synthesis of gold nanoparticles (AuNPs). The concentration of the precursor solution and the extract volume were those of the experimental parameters evaluated. For concentration analysis, HAuCl_4_ solution was varied in 3 mM, 5 mM, and 7 mM, while the extract and precursor volumes used were 0.25 mL for the three first experiments. Then, in a second set of experiments, the volume extract was varied from 0.25 to 1 mL against 1 mL of HAuCl_4_ solution at 7 mM. All the synthesis reactions were carried out at room temperature (~25 °C).

A purification process was then carried out and liquid samples containing the AuNPs were subjected to centrifugation at 10,000 rpm for 15 min. The nanoparticles were then precipitated and the supernatant containing extract residues and unreacted Au ions were removed. Distilled water was then added and the nanoparticles were redispersed using an ultrasonic bath for 10 min. The centrifugation and redispersion process were repeated 3 times to obtain clean nanoparticles for characterization and application.

### 2.5. Characterization of AuNPs

The AuNPs were characterized using six different techniques: Ultraviolet-Visible spectroscopy (UV-Vis) analysis was performed using a UV-6000 spectrophotometer (METASH Instruments Co. Ltd., Shanghai, China), to determine the presence of nanoparticles. The spectra were recorded from 300 nm to 800 nm using quartz cuvettes.

The crystalline nature and crystal size were evaluated by X-ray Diffraction, employing an Ultima IV diffractometer (Rigaku Corporation, Tokyo, Japan). The radiation used was Cu Kα and the 2θ degree range was from 20 to 80°.

The morphology and particle size were analyzed in a Hitachi SU8230 cold-field emission scanning electron microscope (Hitachi Ltd., Tokyo, Japan) with an accelerating voltage of 1 kV.

Hydrodynamic particle size using dynamic light scattering (DLS) and zeta potential of the AuNPs were analyzed with a Zetasizer Nano-SZ (Malvern Panalytical, Worcestershire, United Kingdom) using a dispensable folded capillary cell (DTS1070) for the zeta potential.

Finally, the organic compounds involved in the reduction and stabilization of the AuNPs were suggested based on the functional groups determined by the FT-IR spectroscopy technique, using a Spectrum Two FT-IR Spectrometer (Perkin Elmer, Waltham, MA, USA). The FT-IR spectra were recorded using a wavelength from 400 to 4000 cm^−1^ on dried samples.

### 2.6. Electrochemical Measurements

All electrochemical measurements were performed using a boxer connector as the interface between the screen-printed electrodes (SPE) and the Bio-Logic VP-50 potentiostat (Bio-Logic Science Instruments, Seyssinet-Pariset, France) using EC-Lab software. Commercially available screen-printed carbon electrodes, modified with carboxyl groups functionalized multi-walled carbon nanotubes (CNT-SPE, Type: 110-CNT) from DropSens (Oviedo, Asturias, Spain) were used. The miniaturized electrochemical cell consisted of MWCNT-COOH/Carbon, Carbon and Silver, as working, auxiliary and reference electrodes, respectively.

**Figure 1:**
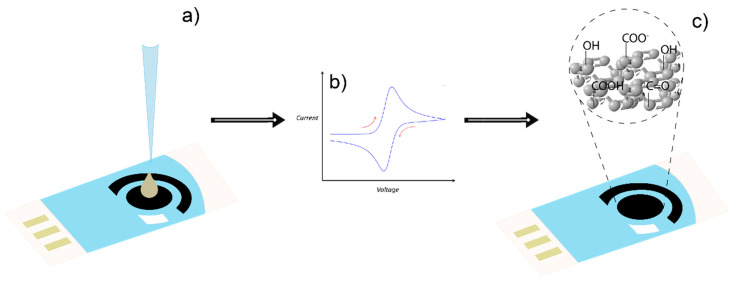
Electrochemical oxidation of the CNT surface on SPE. (**a**) Application of a H_2_SO_4_ drop on the CNT, (**b**) cyclic voltammetry for electrochemical oxidation, and (**c**) oxidized surface of the CNT on the SPE.

In all cases, the screen-printed CNT surface of the working electrode was first modified using electrochemical oxidation performed by cyclic voltammetry (CV) from −1.0 V to +1.0 V at 100 mV/s for 100 cycles (CNT-SPE/A) on a drop (50 μM) of H_2_SO_4_ 0.5 M (Figure 1) [36].

The electrochemical oxidation procedure is intended to generate hydroxyl, carbonyl, and carboxyl moieties, which are relevant since they can be involved in hydrogen bonding and/or covalent binding with other active functional groups, i.e., those present in the molecules of the active compounds of diverse plants, algae, fruits, herbs, or fungi. To observe the enhanced response of the electrode after the oxidation of CNT, measurements using different glucose concentrations by CV were performed and the electrochemical profiles of it between CNT-SPE and CNT-SPE/A were compared.

After the working electrode activation, further modification was performed using the drop-casting method [37]. The synthesized AuNPs were dispersed by sonication for 5 min and then the working electrode was modified by casting a 20 μL drop of AuNPs on it. Finally, it was protected from the air, to avoid contamination, then dried for 5, 24, 35, and 72 h, to find the best length of time for the AuNPs absorption, which was analyzed by cyclic voltammetry (CV) in the presence of Sorensen buffer and different concentrations of glucose.

**Figure 2:**
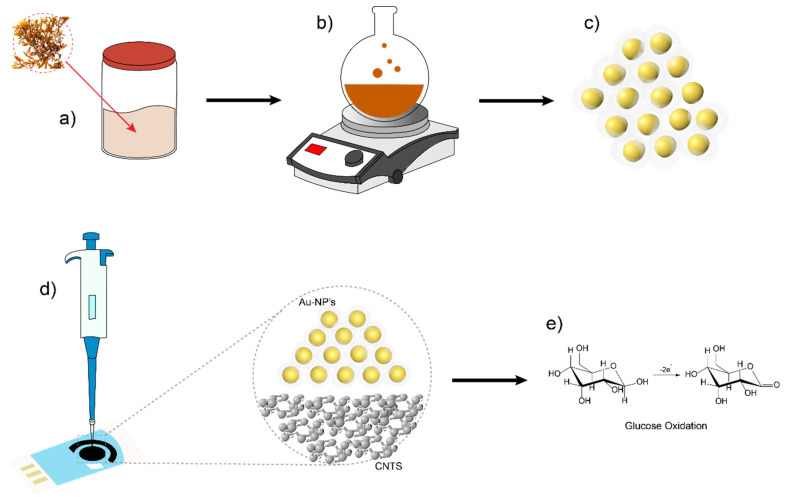
Electrode modification pathway by drop-casting technique using green synthesis AuNPs from Ssp for the assembly of a sensor with carbon nanotubes-screen printed electrodes (CNT-SPE) for glucose detection and quantification. (**a**) Extract of *Sargassum* sp. by infusion method. (**b**) Green synthesis of AuNPs using Ssp extract. (**c**) AuNPs with an organic capping from Ssp compounds. (**d**) Drop-casting of AuNPs on CNT-SPE electrode and their interaction. (**e**) Semi-reaction of glucose oxidation on the CNT-SPE modified with AuNPs.

In this way the electrode was modified and the sensor assembled allowing the binding of the active functional groups present in the oxidized CNT-SPE and the active molecule compounds in the shell of the AuNPs through hydrogen bonding and/or covalent bonding. Overall, using the gold catalytic activity is intended to enhance the measuring of different types of inorganic and/or organic molecules that could affect human health in different media including various foodstuffs. A scheme of the sensor is shown in Figure 2.

To measure the glucose content in food, a 50 μL droplet of Sorensen buffer pH 7 was placed on the CNT-SPE with and without AuNPs in the working electrode, to obtain the background response analyzed by CV, in a potential window of −1 V to +1 V, for two cycles, with a 50 mV/s scan rate. Then, a glucose sample diluted in the buffer (at different concentrations) was analyzed under the same conditions to obtain a calibration curve for anodic current vs glucose concentrations. For food sample analysis, a 50 μL drop of orange juice (a mixture of orange fruit, water and a known quantity of glucose, diluted in Sorensen buffer to reach a concentration of 5 mM) was placed on the electrochemical sensor and analyzed under the same conditions.

## 3. Results and Discussion

### 3.1. Characterization of Gold Nanoparticles

Gold nanoparticles (AuNPs) can be identified by UV-Vis spectroscopy since they exhibit the phenomenon of surface plasmon resonance (SPR), showing an absorption band. For AuNPs, this band is observed between 500 and 600 nm. The position, intensity and shape of the band are based on the size, morphology, and concentration of the nanoparticles.

**Figure 3:**
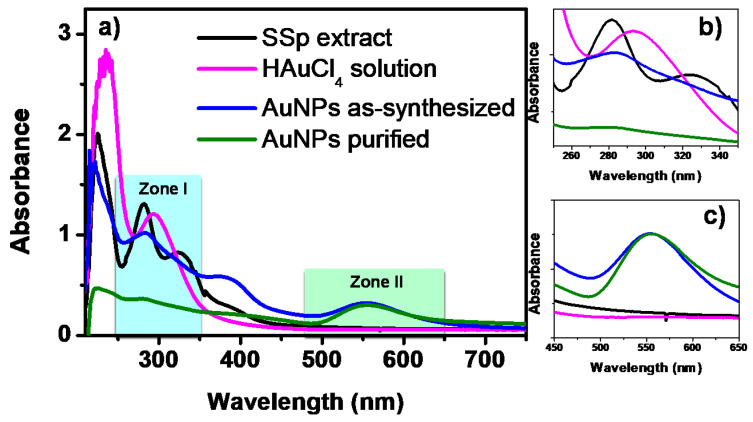
(**a**) UV-Vis analysis of the precursor salt, Ssp extract, and AuNPs before and after the purification process. (**b**,**c**) UV-Vis spectra of the zones I and II obtained from the (**a**).

Figure 3a shows the UV-Vis spectra of the Ssp extract, the HAuCl_4_ precursor solution and the AuNPs before and after the purification process. In this analysis, the presence of AuNPs and the interaction between organic compounds and the HAuCl_4_ aqueous solution can be demonstrated. Two areas of special interest can be noted, named as zone I and zone II corresponding to 200–400 nm and 480–650 nm ranges, respectively. Figure 3b shows a zoom of zone I in which the changes in the signals of the SSp extract and the gold salt are evident, as a result of the AuNPs synthesis. The spectrum of the Ssp extract shows bands located at 226 nm, 282 nm, and 327 nm, corresponding to the phenolic compounds from the Ssp extract. The spectrum of the HAuCl_4_ aqueous solution shows a signal at 295 nm corresponding to Au^3+^, which has already been previously reported [38,39]. Once the synthesis of the AuNPs is carried out, the spectrum shows signals located at 222 nm, 284 nm, and 385 nm, which are attributed to organic compounds and the possible presence of unreacted gold salt. The elimination of residues of the precursor salt and organic compounds was carried out with the purification of the AuNPs. As can be seen, there is only one low intensity band, centered at 279 nm that corresponds to the organic compounds stabilizing the nanoparticles. Therefore, the absence of the absorption band at 295 nm corroborates that there are no Au^3+^ ions residues in the purified AuNPs sample used for the sensing experiments. Figure 3c shows a zoom of the zone II between 480 nm and 650 nm. As can be seen, the spectra corresponding to the AuNPs, before and after the purification process, present the absorption band that corroborates the synthesis of gold nanoparticles. It should be noted that there are no significant differences in the position, shape, and width of said absorption band. This means that the size, shape and size distribution are very similar in both samples. Then, the purification process does not affect the concentration and the physical characteristics of the nanoparticles.

**Figure 4:**
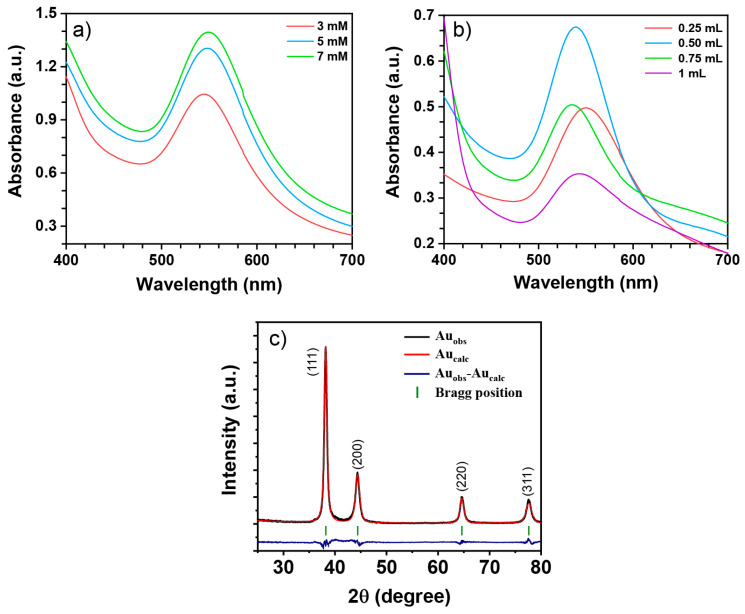
UV-Vis analysis of AuNPs varying (**a**) HAuCl_4_ solution concentration and (**b**) *Sargassum* sp. extract volume, (**c**) Rietveld refinement XRD pattern of Au nanoparticles.

Figure 4a shows the UV-Vis spectra of AuNPs, varying the concentration of the precursor salt. The concentrations evaluated for green synthesis AuNPs were 3, 5, and 7 mM. As can be seen, the three spectra show the absorption bands that corroborate the synthesis of AuNPs [40]. As the salt concentration increases, the intensity of the absorption band also grows, meaning an increase in the concentration of nanoparticles. On the other hand, no significant changes were observed in the position of the absorption band of the three spectra (549 nm). These results suggest that the average size of the nanoparticles is very similar in all samples. Therefore, the samples with a 7 mM concentration of HAuCl_4_ were selected to evaluate the rest of experimental parameters.

The volume of Ssp extract is shown in Figure 4b. The results depend directly on the type and composition of the extract. The spectrum corresponding to 0.25 mL of Ssp extract has an absorption band at 549 nm. When the volume of the extract was increased to 0.5 mL, the absorption band showed a significant increase in intensity. In addition, the position of the band shifted (to 538 nm), suggesting a reduction in the average nanoparticle size. When 0.75 mL of Ssp extract was used, the AuNPs absorption band decreased significantly, meaning a decrease in the concentration of nanoparticles. The position of the absorption band remains unchanged (538 nm), meaning that the average size of the AuNPs is similar to that of the previous sample. The intensity of the absorption band continued to decrease when 1 mL of Ssp extract was used. In this case, the signal is centered at 543 nm, which represents an increase in the size of AuNPs. Moreover, it should be noted that the absorption band broadened in this spectrum, suggesting a wider size distribution. There are some reports in which the increase in the volume of extract improves the characteristics of the nanoparticles [41]. However, in several studies the opposite has been reported [42,43,44,45]. The increase in the extract volume leads to the synthesis of nanoparticles with several morphologies. This may be due to the poor control of the reaction as several compounds were involved. Moreover, if there are not enough stabilizing agent compounds to cloister the nanoparticles, uncontrolled growth can occur, leading to nanoparticle synthesis outside the nanometric scale. Therefore, the sample corresponding to nanoparticles synthetized with 0.5 mL of Ssp extract was selected for subsequent characterization and electrochemical analysis.

X-ray diffraction (XRD) was performed to confirm the crystalline nature of the synthesized AuNPs. Figure 4c shows the XRD pattern, in which there are four reflections, at 38.21°, 44.61°, 64.62°, and 77.62° corresponding to (111), (200), and (311) crystallographic planes, respectively. The positions of all the peaks coincide with the face-centered cubic structure of metallic gold (JCPDS 04-0784) with Fm-3m space group. Rietveld’s refinement XRD method using Profex software [46] was carried out in order to investigate the lattice parameters and crystalline size. As can be observed from the plots generated by the Rietveld’s refinement of the sample, that the observed and the calculated diffractograms are in good agreement. The lattices parameter obtained from Rietveld analysis was 4.0761 Å, which is in accordance with the gold structure data reported in the literature. A Williamson–Hall plot was used to estimate the crystalline size using the FWHM of all diffraction peaks. The average crystallite size was estimated to be 13.80 ± 1.79 nm. These results confirm the presence of gold as the unique crystalline phase. No impure peaks were detected, meaning that the synthetized nanoparticles are very pure and explaining the role of organic compounds from Ssp extract in the synthetization of nanoparticles.

**Figure 5:**
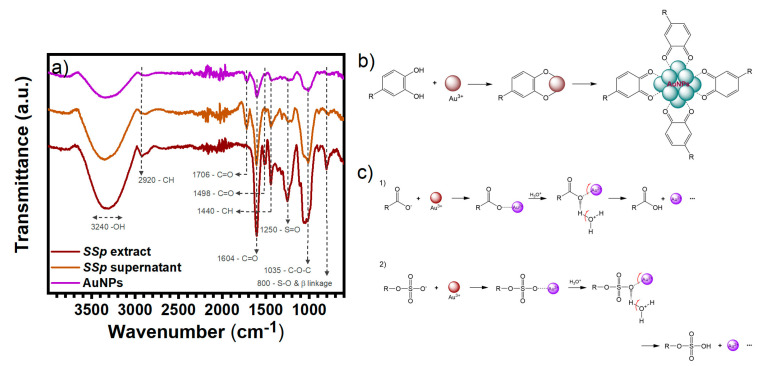
(**a**) FT-IR analysis of AuNPs, and *Sargassum* sp. extract before and after the synthesis reaction. (**b**) Scheme of the reaction mechanism for AuNPs in the presence of phenolic compounds and (**c**) Scheme for the reaction mechanism for AuNPs in presence of (1) alginic acid and (2) fucoidan.

FT-IR analysis was carried out to determine the organic compounds involved in the AuNPs synthesis. The spectra of the Ssp extract (wine line), the supernatant obtained after washing (brown line) and the synthesized AuNPs (purple line) are shown in Figure 5a. The Ssp extract shows several characteristic bands. The signal located at 3240 cm^−1^ corresponds to the O-H symmetrical stretching vibration. Bands at 2920 cm^−1^ and 1440 cm^−1^ correspond to C-H symmetric stretching and C-H bending, respectively. The bands located at 1604 cm^−1^ correspond to C=O from polysaccharides and 1498 cm^−1^ correspond to C=O from aromatic bonds. The band around 1250 cm^−1^ corresponds to S=O asymmetrical stretching vibration from fucoidans. The band around 1035 cm^−1^ is related to C-O stretching from ester groups. Finally, bands at 894 cm^−1^ and 800 cm^−1^ show the presence of sulphate groups, since they can be attributed to S-O stretching. All these characteristic bands correspond closely to phenolic and polysaccharide compounds. The supernatant studied showed similar bands with a decrease in some intensities and the bands corresponding to O-H, C-H, C=O, C-O, and S-O bonds still present. On the other hand, the spectrum of AuNPs showed fewer bands and different intensities. Only signals located at 3220 cm^−1^, 2920 cm^−1^, 1706 cm^−1^, 1604 cm^−1^, 1440 cm^−1^, 1182 cm^−1^, 894 cm^−1^ are still present, corresponding to O-H stretching, C-H stretching, C=O stretching, C=O from polysaccharides, C-H stretching, C-O stretching, and S-O stretching, respectively.

Significant changes in peak intensities were observed when comparing the spectra. In the supernatant, the band at 1182 cm^−1^ is clearer, corresponding to C-O stretching. Likewise, the intensity of the band at 1706 cm^−1^ is augmented in contrast with the diminishing band at 1604 cm^−1^, both corresponding to the C=O stretching, due to the interaction of an electron accepting group, such as gold or trough intramolecular hydrogen-bonded molecules of the extract after reaction with the gold salt. This explains why this frequency is of a higher intensity in the supernatant than in the AuNPs. Meanwhile, the band at 1498 cm^−1^, corresponding to the C=O aromatic bond, disappeared. The similarities in the spectra show that many of the molecules in the extract did not form a bond with the nanoparticles and remained dispersed in the medium. For the AuNPs, the decrease in intensities could be related to a decrease in the concentration of the extract molecules; however, these results confirm that the phenolic compounds and polysaccharides in Ssp are involved in the gold reduction and its stabilization, resulting in the formation of nanoparticles with an organic capping.

The exact mechanism in the green synthesis of nanoparticles is difficult to determine, since there are many organic compounds present in the system. In addition, several compounds may intervene in the reduction and stabilization of the nanoparticles at the same time. The idea that phenolic compounds and polysaccharides actively participate in AuNPs synthesis, arises because a significant amount of these compounds can be observed from FT-IR. This is supported by different reaction mechanisms explained elsewhere [39,47,48,49].

The reaction mechanism of AuNPs from phenolic compounds present in the Ssp is shown in Figure 5b. First, the ortho-dihydroxyl group from the phenolic compound chelates Au^3+^ ions, to form a five membered chelate ring. Then, due to the high oxidation-reduction potential of Au^3+^ ions, the chelated ortho-dihydroxyl groups are oxidized to quinones with a concomitant reduction of the Au^3+^ ion to Au^0^. Finally, the closest Au^0^ atoms collide to form the nanoparticles (AuNPs) and these are then stabilized by other phenolic compounds, as well as the quinones. In other words, a 2-electron oxidation takes place, from the adjacent hydroxyl groups to form a quinone, Au^3+^ is reduced to Au^0^ and the negative charged carbonyl groups help to stabilize the NPs surface, forming part of the AuNPs capping.

The reaction mechanism for AuNPs using polysaccharides such as the present using the Ssp is shown in Figure 5c. As described elsewhere [13], there are two major polysaccharide constituents: alginate which is the calcium, sodium or magnesium salt from alginic acid which is composed of (1→4)-α-L-guluronate and β-D-mannuronate residues and fucoidan formed mainly by (1→3)-α-fucose with sulphate groups on some residues at position 4. The reductor character from both compounds is given by their functional groups end, for alginate a carboxylate ion (-COO^−^) after salts dissociate in the aqueous media, and for fucoidan from the sulphate group. First, the negatively charged oxygen, from both carboxylate and sulphate, interacts with the Au^3+^ ions and, because of their free electrons, is reduced to Au^2+^ ions. Then, the presence of hydronium in the media serves for a electrophilic attack to the carbonxylate/sulphate of the polysaccharide, detaching the Au^2+^ ion and reducing the molecules to carboxylic acid in the case of alginate (alginic acid) and hydrogen sulfite in the case of fucoidan. Finally, the reduction of the Au^2+^ to Au^0^ is carried out in the manner described to form an Au^1+^ ion, that initially undergoes the same process until the Au^0^ atom is formed and then collides with other Au^0^ atoms and is stabilized with the nearest organic molecules.

Furthermore, it is important to highlight that by either of these two mechanisms, it has been reported that part of the Au^3+^ atoms are not completely reduced, remaining in an intermediate oxidation state (Au^+^). Au^+^ atoms are arranged on the surface of the nanoparticles, acquiring a positive surface charge [50,51].

**Figure 6:**
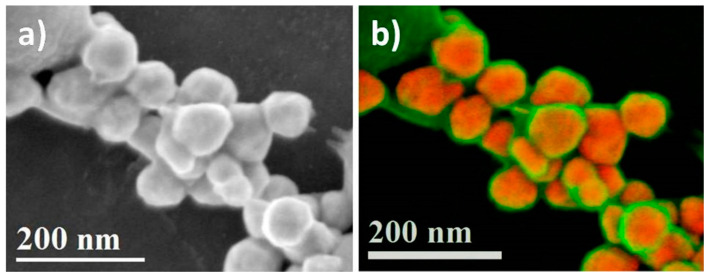
(**a**) SE-SEM and (**b**) BSE+SE SEM images showing the size and morphology of AuNPs synthesized with Ssp extract.

Figure 6a shows the secondary electron-scanning electron microscopy (SE-SEM) micrograph of the synthesized AuNPs. As can be seen in this micrograph, there is a high concentration of nanoparticles, of various morphologies, and an organic compound that is coating the nanoparticles is clearly visible. These results confirm the ability of the Ssp extract to synthesize AuNPs. Figure 5b shows a color look-up table (LUT) image formed by mixing the SE-SEM and backscattered electron-scanning electron microscopy (BSE-SEM) micrographs. It is clear from the image that all the nanoparticles are coated by an organic compound (green) from the Ssp extract, and the characteristic shapes of the nanoparticles (red) are shown in greater detail. The catalytic activity and selectivity of the nanoparticles depend on the shape, morphology, and size. The particle size ranges from 80 to 100 nm, with several polyhedral shapes. The presence of various polyhedral shapes can be attributed to the different organic compounds involved in the reaction described. For face-centered cubic metals, single-crystal seeds tend to evolve into single-crystal products with polyhedral shapes, the so-called Wulff polyhedrons, depending on the ratio of growth rates along the (111) and (100) facets, which is determined by the type and concentration of the capping agent [52]. Most nanoparticles are shaped like the Wulff polyhedron (truncated octahedron); however, some nanoparticles were obtained with different shapes that can be attributed to the equilibrium condition never being reached during synthesis. The surface energies are different due to anisotropic interactions of the capping agent [53].

**Figure 7:**
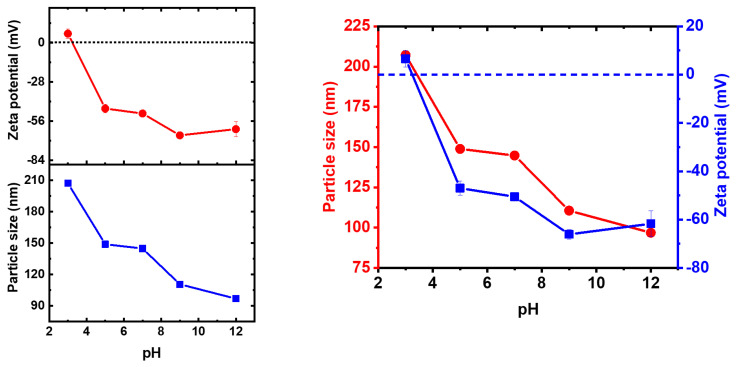
DLS graph and zeta potential (ζ) of AuNPs as a function of pH, ranging from 3 to 12.

For a colloidal stability analysis of the AuNPs, the pH of the samples was varied, and the changes of the surface charge and particle size was studied. The ζ values of surface charge for the AuNPs switch when the pH increases. The nanoparticles show excellent stability at pH values between 12 and 5. The measured potential was −62 mV, −65 mV, −51 mV, and −47 mV for pH values of 12, 9, 7, and 5, respectively. When a pH of 3 was evaluated, a positive potential of 6 mV was obtained. This positive potential indicates that the nanoparticles are less stable and may lead to their agglomeration, as a result of the Van der Waals attractive forces [54]. The isoelectric point was 3.2, which is lower than those reported in thiol-gold samples [55], suggesting the effect of organic layer on the surface net charge. Therefore, the organic layer provides stability to the AuNPs. The hydrodynamic diameter distribution and zeta potential (ζ) of the AuNPs as a function of pH were measured and the results are shown in Figure 7. Dynamic light scattering (DLS) data for the AuNPs showed a hydrodynamic diameter average size of 96 nm at pH 12, and it was increased as the pH was decreasing, until 207 nm at pH 3; these values indicate a slight agglomeration of the particles. As is known, DLS shows larger average size values compared to SEM, when an organic layer is around the particles.

### 3.2. Working Electrode Activation Analysis

The present study investigated the efficiency of the organic capped AuNPs deposited on the surface of carbon nanotubes-screen printed electrodes (CNT-SPE) as a portable glucose electrochemical sensor. However, electrochemical activation of the CNT-SPE is needed to partially oxidize the surface of the CNT-SPE and to expose the functional groups present on CNT (−OH, C=O and COO−), for later modification of the electrode using AuNPs. For observing the changes in the electrochemical profile after modification, Sorensen buffer and glucose were used.

**Figure 8:**
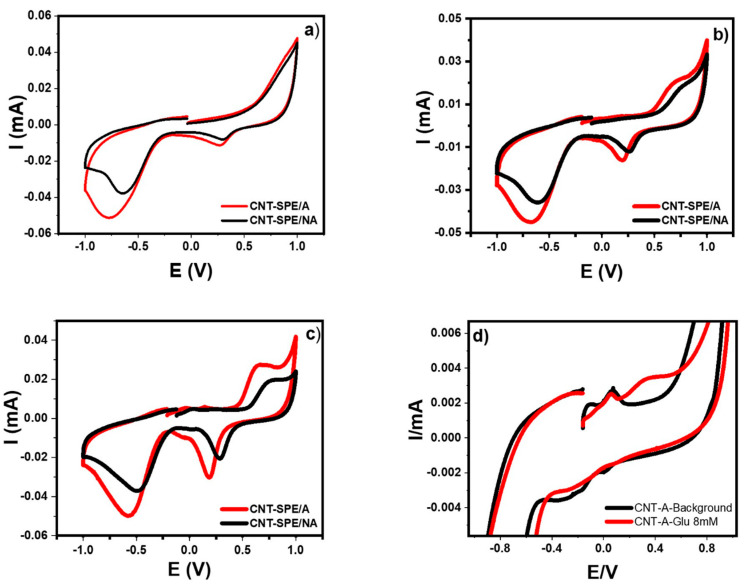
Cyclic voltammetry of (**a**) Sorensen buffer pH7, (**b**) Glucose 8 mM, (**c**) Glucose 4 mM on CNT-SPE electrochemically activated (CNT-SPE/A) and without activation (CNT-SPE/NA), and (**d**) comparison between Sorensen buffer (pH = 7) and glucose 8 mM on CNT-SPE/A.

Figure 8 shows the overlapped voltammetry response made by cyclic voltammetry (CV) of both electrochemical sensors, CNT-SPE was electrochemically activated (CNT-SPE/A) and non-activated (CNT-SPE/NA) in the presence of the buffer at various glucose concentrations.

**Scheme 1:**
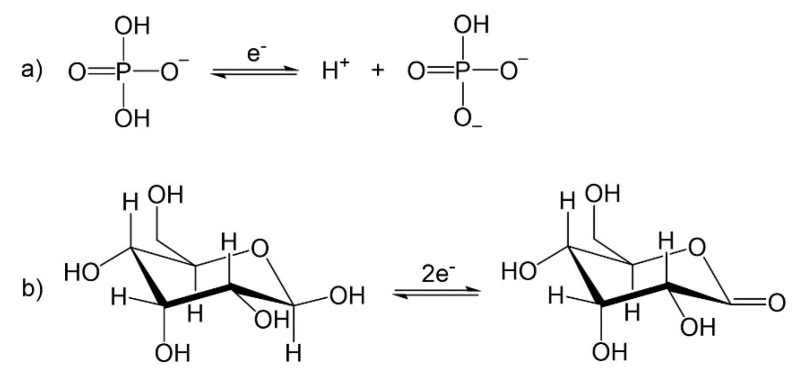
Reactions involved in the oxidation and reduction of (**a**) Sorensen buffer and (**b**) glucose to sorbitol.

In all cases, a reduction signal at around 170 mV was seen in the presence of glucose and at 271 mV when only the buffer was present. These two signals are related to the chemical equations presented in Scheme 1, respectively.

Analysis of the CV from the oxidized electrode of the Sorensen buffer and 8 mM glucose (Figure 8a,b) shows that the current is higher and there is a shift towards more negative potential when the electrode is activated. This is because there are more functional groups with a negative charge, so all the studies employed a previously electrochemical activation, which will keep these groups available and a larger surface area, facilitating the electronic transfer, that may explain the potential displacement and the increase in the current. On the other hand, a new oxidation signal is observed around 650 mV when glucose is present in the analysis. This increase is inversely proportional to its concentration as seen when analyzing the 4 mM response (Figure 8c). This indicates an electrode passivation in larger quantities of glucose. It is worth mentioning that in a higher oxidation current, an increase in the reduction current is also generated, and so the reduction signal is related to the products of glucose oxidation.

Figure 8d shows a close-up of the oxidation and reduction peaks present on the CNT-SPE/A. In the black line, two oxidation signals (−95 mV and 68 mV) and two more of reduction (8 mV and −206 mV) related to the background (buffer) can be observed. When an appropriate volume of glucose is added to obtain a concentration of 8 mM (red line), the oxidation and reduction peaks of buffer (except the signal in 68 mV) disappear and a new peak is observed at 346 mV, associated to the response of glucose oxidation on CNT-SPE/A.

### 3.3. Glucose Sensor Platform Based on AuNPs + CNT-SPE Assembling

Once the CNT-SPE was electrochemically activated, AuNPs were deposited on the electrode. To check that the assembling of the electrochemical sensor was successful, a microscopic analysis of the CNT-SPE/A after modification with AuNPs using SEM was performed.

**Figure 9:**
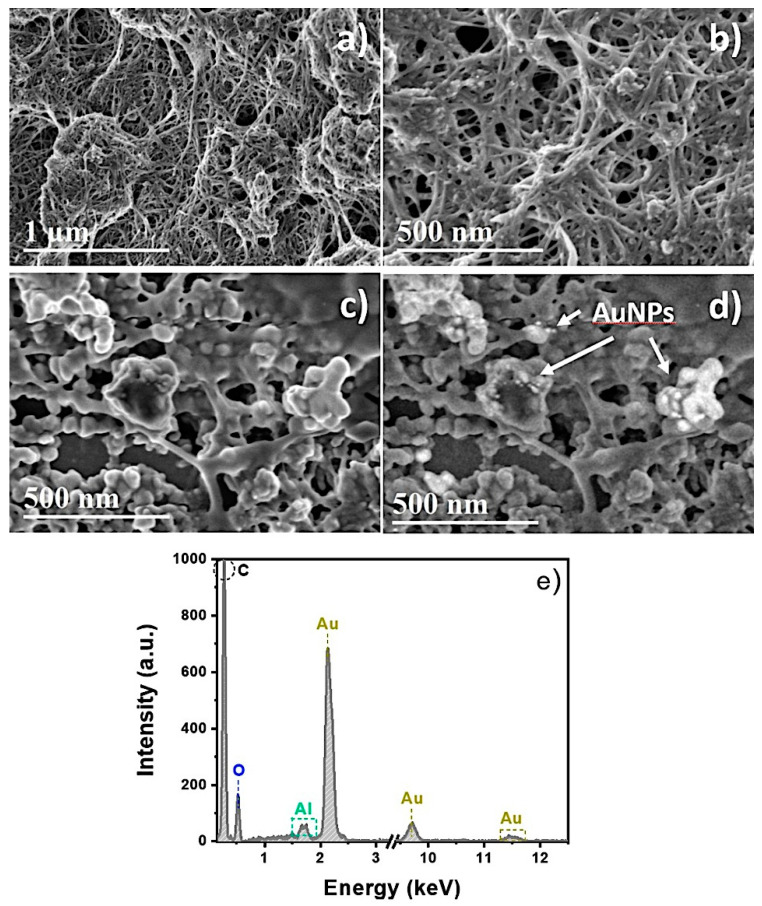
(**a**,**b**) SE-SEM images of commercial CNT-SPE, (**c**,**d**) SE-SEM images of the commercial CNT-SPE modified with AuNPs, and (**e**) EDS spectrum of commercial CNT-SPE and AuNPs.

Figure 9a,b shows the SE-SEM micrographs of the commercial CNT-SPE. The micrographs show a dense mesh-like deposit of CNT of irregular sizes. The CNT-SPE looked like porous carbon electrodes because of the disordered arrangements of the CNT over the SPE surface. The SE-SEM micrograph of the commercial CNT-SPE modified with AuNPs of Figure 9c shows that some CNTs are visible on the surface, indicating that the electrode still has a certain porosity. Likewise, Figure 9d shows the mixed SE and BSE-SEM micrograph of the commercial CNT-SPE modified with AuNPs. The bright dots are the AuNPs, distributed uniformly over the surface of the electrode. Figure 9e shows the energy dispersive spectroscopy (EDS) spectrum of the commercial CNT-SPE modified with AuNPs. The EDS spectrum (Figure 7e) shows the presence of Au, O, and C (the Al signal is associated with the sample holder), which comes from the sample.

### 3.4. Glucose Detection Analysis

**Figure 10:**
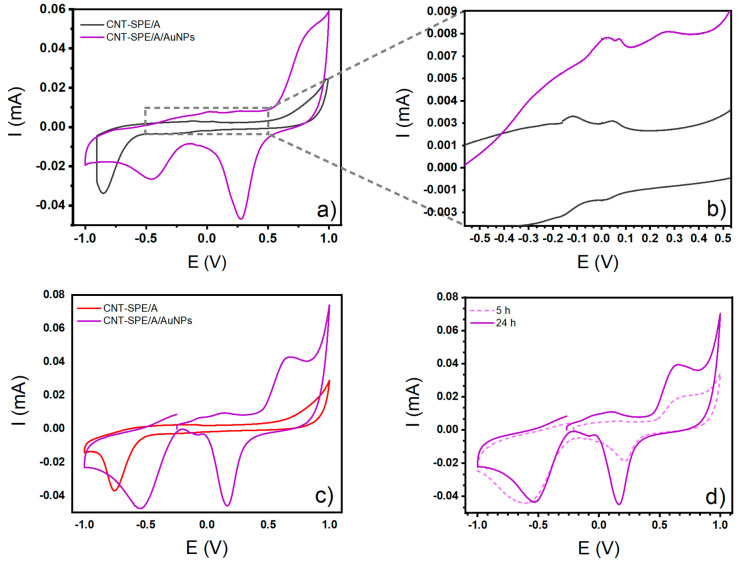
Cyclic voltammogram curves of (**a**) Sorensen buffer (support electrolyte @ pH = 7) on CNT-SPE/A and CNT-SPE/A/AuNPs, (**b**) close up of the oxidation peaks of CNT-SPE/A and CNT-SPE/A/AuNPs on the support electrolyte, (**c**) glucose 8 mM studied on CNT-SPE/A and glucose 8 mM on CNT-SPE/A/AuNPs after 24 h modification, (**d**) glucose 4 mM on CNT-SPE/A/AuNPs after 5 and 24 h modification.

Figure 10 shows food additive electrochemical sensing analysis with glucose using the oxidized working electrode, after AuNP modification. Figure 10c shows the overlapped CV analysis of glucose 8 mM using the activated electrode (CNT-SPE/A) and its modification using AuNPs (CNT-SPE/A/AuNPs). A drastic change in the electrochemical behavior of the glucose is observed; there are three oxidation signals, at −58 mV, 145 mV, and 659 mV, that are not present in background, confirming the good interaction of glucose with AuNPs.

When observing the Sorensen buffer analysis against that of 8 mM glucose (Figure 10a,c, respectively), the mechanism of electrooxidation of glucose can be described as follows. At first, when CV from support electrolyte on CNT-SPE/A and CNT-SPE/A/AuNPs were analyzed, an oxidation peak (shown like two small humps, close together in Figure 10b) is seen in both cases, attributed to the adsorption of the hydroxyl groups present in the medium. Following the sweep in the anodic direction, a second signal appears, but only in the analysis with CNT-SPE/A/AuNPs, corresponding to the oxidation of gold. The same signal is seen to be reduced when the sweep is made in the cathodic direction. When glucose was analyzed, the two small peaks seen in the background and attributed to the active compounds adsorbed, were still present. These correspond to hydroxyl, carbonyl, and carboxylic groups, now belonging to the hemiacetal group of glucose, which is linked to the surface of CNT-SPE/A/AuNPs. This can give an insight into the system for surface adsorbed substances.

**Scheme 2:**
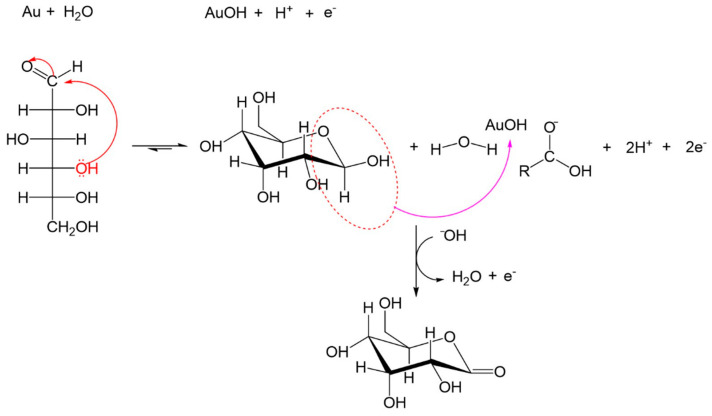
Mechanism proposed for glucose electrooxidation.

In a second step, the interaction of hemiacetal group of glucose and the adsorbed hydroxyl make oxidation possible, with the H bound to C1 being the first to suffer the oxidation on AuNPs, producing the radical species, which is reflected in the voltammogram to glucose on CNT-SPE/A/AuNPs in 645 mV. The radical species are also oxidized to produce gluconolactone, where another electron is transferred to the electrode. Then gluconolactone is desorbed to the electrode-forming sodium gluconate in the same way it is reduced backwards. This whole process involves the transfer of two electrons, as can be observed in Scheme 2.

Figure 10d shows the CV of glucose 4 mM on CNT-SPE/A/AuNPs after 5 and 24 h of drying. A higher current was found when the modification was carried out over 24 h. Therefore, all subsequent modifications were developed in this way. It is worth mentioning that while it is true that longer times may lead to higher currents, it is also true that elevated modification times affect the efficiency of the method, making it slow and less functional. It is also a fact that AuNPs have an organic capping from Ssp. compounds, which at higher times can have intermolecular interactions, and lead to agglomeration in the CNT-SPE surfaces, reducing the active area and therefore the efficiency of the sensor.

### 3.5. Effect of Scan Rate on Glucose Detection Analysis

**Figure 11:**
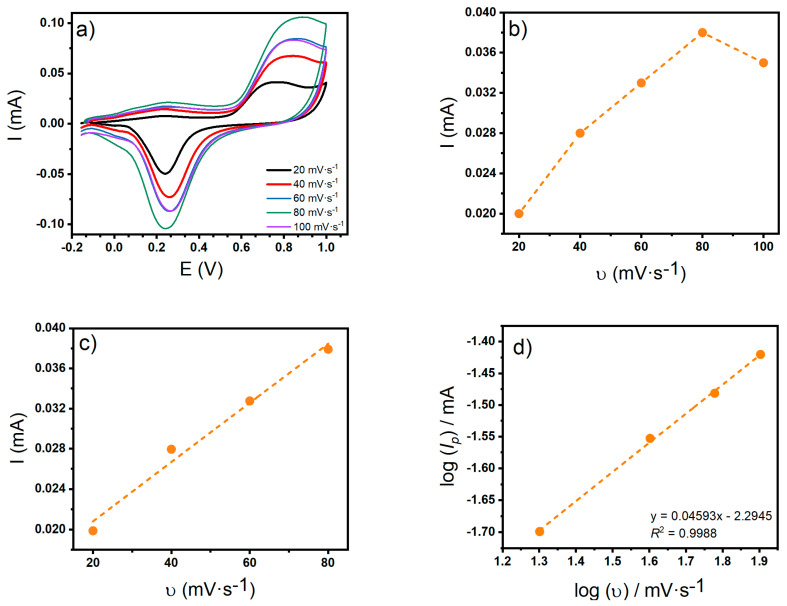
(**a**) Cyclic voltammogram at different scan rates, (**b**) plots of anodic peak currents versus scan rate, up to 100 mV-s^−1^ (**c**) anodic peak currents versus scan rate up to 80 mV-s^−1^ and (**d**) logarithmic plots of anodic peak currents versus scan rate. All the voltammograms correspond to glucose 10 mM in Sorensen buffer pH 7 on CNT-SPE/A/AuNPs.

An interesting aspect in the electrochemical analysis is the variation in potential and current peak intensities generated in CV with the change of sweep rate (20–100 mV s^−1^). These are parameters that can help to characterize physicochemical aspects of the reaction. Figure 11a shows the CV voltammograms overlapped at different sweep rates with the presence of 10 mM glucose on CNT-SPE/A/AuNPs. Figure 11b presents the dependence of the anodic peak current at different sweep rates, up to 100 mV s^−1^, showing a linear behavior up to 80 mV s^−1^ (Figure 11c). There is also a linear relationship between $\log\left(I_{p}\right)$ and log(v), seen in Figure 11d, given a slope value of 0.4593 and a relation coefficient of R^2^ = 0.9988.

**Figure 12:**
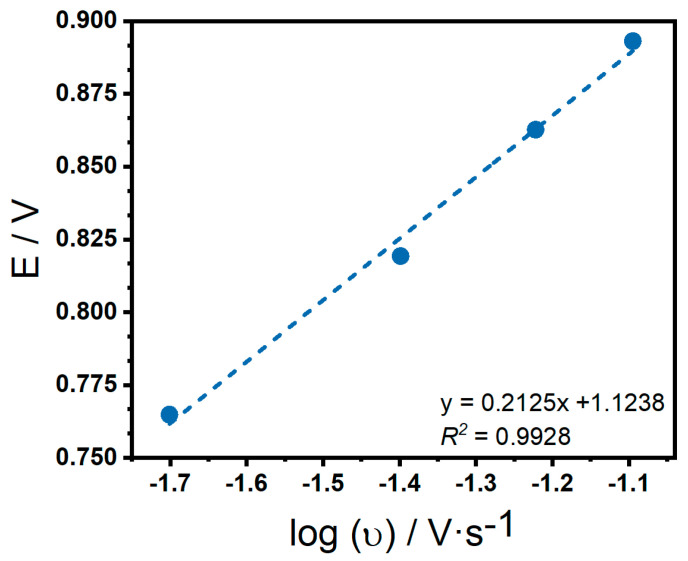
Linear regression to anodic potential against logarithmic sweep rate of glucose 10 mM on CNT-SPE/A/AuNPs in Sorensen buffer pH 7.

The reversibility of the system was studied by analyzing oxidation and reduction current peaks. In Figure 12 the linear regression between *E_p_* and log(v) shows a good linearity for a slope of 0.2125 and a relation coefficient of R^2^ = 0.9928.

As mentioned earlier, scan rate variations give an insight into the electrochemical process. From the linear fitting from $\log\left(I_{p}\right)$ and log(v), it is observed that its slope value is close to 0.5, confirming that the electrode process is diffusion controlled [56]. The fit of *E_p_* and log(v), suggests that this is an irreversible process, according to the expression from Laviron theory in Equation (1) [57]:

(1) $$Ep=E^{0}+\left(2.303RT/\alpha nF\right)\log\left(RTk^{0}/\alpha nF\right)+\left(2.303RT/\alpha nF\right)\log\left(v\right),$$

where α is the transfer coefficient, $k^{0}$ is the standard heterogenous rate constant for the reaction, *n* is the number of electrons, v the sweep rate and $E^{0}$ the standard redox potential. The rest of the symbols have their usual meaning. The elevated separation of anodic and cathodic peaks (646 mV) and the inequality in terms of their current intensities, confirm this.

Taking into consideration all the electrochemical analysis, it can be deduced that the whole process of glucose electro-oxidation involves the slow transfer of two electrons (n = 2) in an irreversible diffusion control process. This system was determined as irreversible so the transfer coefficient can be calculated using the equation proposed by Bard-Faulkner [58,59] (Equation (2)) resulting in *α* = 0.22:

(2) $$\Delta E_{p}=E_{p}-E_{p/2}=47.7/\alpha n,$$

where $E_{p}$ is the potential peak and $E_{p/2}$ is the width in the middle of the current peak. In this case, where the α value is below 0.5, we can deduce that the highest percentage of the energy used favors the reduction reaction.

**Figure 13:**
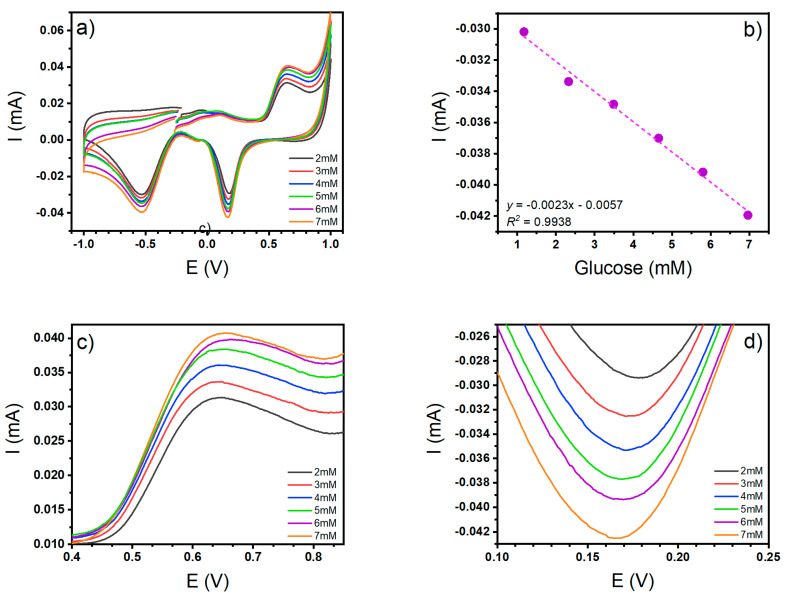
(**a**) Cyclic voltammetry to glucose at different concentrations, (**b**) Calibration curve using the cathodic peak currents, (**c**) Close-up of the oxidation signal, (**d**) Close-up of the reduction signal.

Finally, the analytical measurements for glucose detection on the CNT-SPE/A/AuNPs are seen in Figure 13a, where the cyclic voltammetry response to different concentrations of glucose is graphed. A proportional increase in the concentration of both oxidation and reduction signals was observed with R^2^ = 0.99 and 0.96 to reduction and oxidation signals (Figure 13b). Figure 13c,d show a close-up of the peaks in the oxidation and reduction signals, respectively. This was performed to reinforce the calibration curve analysis.

As can be seen, the reduction signal has the best linear relation, so this is the signal peak that was used for the detection and quantification of glucose. Figure 13b was also used for the limits of detection (LOD) and quantification (LOQ) calculi, which were obtained through the means of a statistical method, where the intercept of the linear relationships LOD =a+3σ and LOQ =a+10σ, was used (where σ is the standard deviation of y-residuals of the linear regression). The calculated LOD was 50 μM and the calculated LOQ was 98 μM for glucose.

### 3.6. Stability, Repeatability, Reproducibility, and Application of the Platform

**Figure 14:**
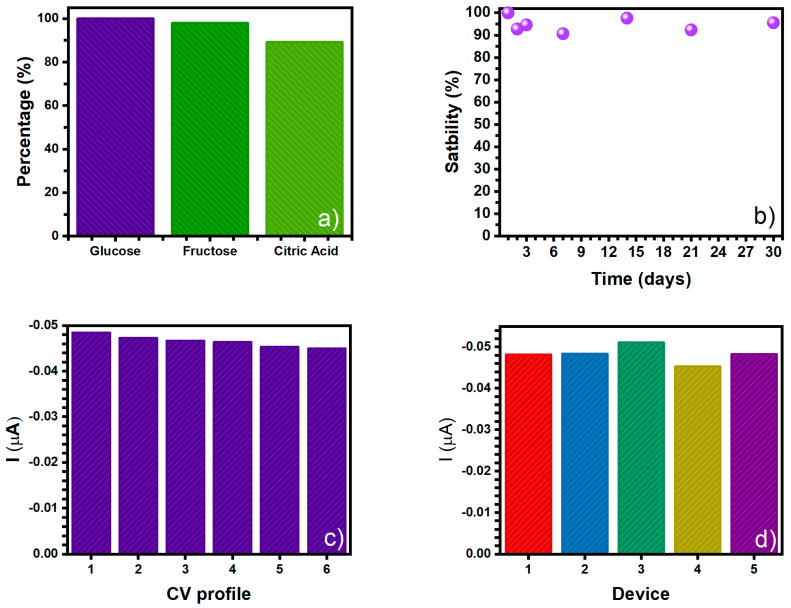
(**a**) Effects of the presence of food additives fructose and citric acid on the CV measurement for 7 mM of glucose in CNT-SPE/A/AuNPs, (**b**) electrochemical response stability of the CNT-SPE electrode after 30 days to 7 mM of glucose, (**c**) evaluation of repeatability, and (**d**) evaluation of reproducibility.

An interference analysis was also made with molecules that could be used as food additives in diverse alimentary products. The CV experiments were performed using 7 mM of glucose and then adding 7 mM of fructose or citric acid in two different experiments. Figure 14a shows the current percentage of the CV glucose profiles in the presence of fructose or citric acid. For the glucose + fructose analysis the reduction peak is 97.92% of the value of the original glucose peak and for glucose + citric acid analysis the reduction peak is 89.13% of the original current intensity. The data demonstrates that the CNT-SPE/A/AuNPs platform was able to determine glucose in the presence of two chemical species, which are commonly found in food products.

A stability analysis was also performed, measuring the reduction peak signal from glucose over time. The sample was measured daily for the first three days and then weekly for a month (approximately 30 days) of elapsed time. The results are shown in Figure 14b. It can be observed that the electrochemical platform (CNT-SPE/A/AuNPs) has a mid-term stability since it can be stored at room temperature for 30 days, with an average loss of 93.93% of the initial current values. The given relative standard deviation (RSD) was 2.51% (*n* = 6) for the reduction peak current of glucose CV profile.

In addition, repeatability, and reproducibility studies of the CNT-SPE/A/AuNPs platforms were carried out. For the former the CNT-SPE was tested using a 7mM glucose sample following its reduction peak current and it was estimated using 6 CV profiles (Figure 14c). When the platform is used and then used again, the anodic current value falls, giving an RSD of 2.65%. In the reproducibility analysis, five different CNT-SPE/A/AuNPs sensing platforms were prepared at the same time and each was tested on a 7 mM glucose sample. In Figure 14d a 4.01% RSD is seen, indicating that the platform has a good reproducibility with the batch of AuNPs synthetized.

**Figure 15:**
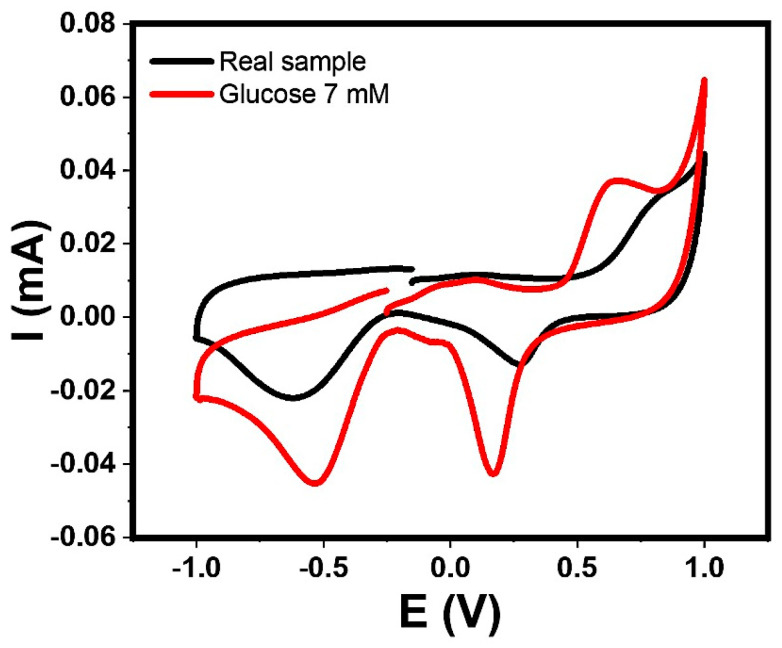
Cyclic voltammogram analysis corresponding to prepared orange juice, and glucose 7 mM. Scan rate: 50 mV/s and window potential: −1.0 to +1.0 V on CNT-SPE/A/AuNPs in Sorensen buffer pH 7.

Finally, when the commercial orange juice sample was analyzed (orange juice with 5 mM of glucose) a reduction signal was observed with a current of 0.0133 mA, and a displaced potential signal in Figure 15. This may be due to the contribution of other compounds present, but when the obtained current is substituted in the equation of the line generated in the calibration curve, the concentration is efficiently correlated.

A comparison was made of the portable electrochemical sensor of the present work (CNT-SPE/A/AuNPs) with the latest glucose electrochemical sensors developed in which LOD is included (Table 1).

## 4. Conclusions

In this paper the feasibility was evaluated of using green synthesis AuNPs with an organic coating in the assembling of a fast, sensitive, low cost and precise electrochemical platform of CNT-SPE electrodes to sense glucose without the use of an enzyme. The fundamentals of the electrochemical process involved were investigated. The main conclusions that can be drawn from this study are:

- It is the first report of green synthesized AuNPs to be used in combination with screen-printed electrodes for analyte sensing, in this case, glucose.
- The green synthesis of AuNPs using the phenolic compounds and polysaccharides obtained from *Sargassum* sp. extract was successful since from UV-Vis surface plasmon from gold is observed and from XRD, gold is the only crystalline phase present, with a crystallite size 13.80 nm. The nanoparticles obtained have an average size of 80–100 nm and an excellent stability in a wide range of pH (from 5 to 12).
- The carbon nanotube-screen printed electrode activated and modified with AuNPs electrochemical sensor obtained (CNT-SPE/A/AuNPs), is an efficient device able to detect small concentrations of glucose (50 μM). This is lower than the response obtained by electrochemical sensors modified without any enzyme employment [45,46], making it a competitive option.
- This sensor is a sensitive, reliable, low-cost platform to measure glucose, since it uses small sample volumes, offering mid-term stability, repeatability, and reproducibility. In both cases low LOD and LOQ were detected.
- The inherent miniaturization of the electrochemical system is easier because of the synthesis method of AuNPs used (green synthesis from *Sargassum* sp.) This creates a synergic effect that gives an analytical method for the detection of glucose in an ecofriendly way. At the same time, it increases sensitivity and specificity due to the presence of gold nanoparticles, which were easily anchored by placing 20 μL on the surface of the working electrode in the screen-printed electrodes.
- For a better understanding of the process involved in the electrochemical performance of the developed sensor, selectivity, interference with other food additives and contaminants, simultaneous detection of hydrogen peroxide, and LOD are areas for further study.

## Figures and Tables

**Table 1:** Comparison of different electrochemical sensors for detecting glucose.

Working Electrode	Type of Cell	Advantage	Disadvantage	LOD
Cu-nanoflower decorated AuNPs-GO Nano Fibers [60]	Conventional 3-electrode cell. Ref ^1^: Ag/AgCl CE ^2^: Platinum	Promising electrochemical biosensor	Uses enzyme, electrospinning and requiring an organic solvent, which is harmful to health and environment	0.018 μM
Three-dimensional graphene foam decorated with Cu-xCu_2_O nanoparticles [61]	Conventional 3-electrode cell: Ref ^1^: Calomel CE ^2^: Platinum foil	Inexpensive, feasible, and non-enzymatic	Mercury content in the reference electrode which makes it toxic	16 μM
Gold layer onto graphene paper [62]	Conventional 3-electrode cell: Ref ^1^: Calomel CE ^2^: Platinum foil	High performance, stability, non-enzymatic	Require additional methods to modification such as furnace or laser Mercury content in the reference electrode which makes it toxic	Down to 2.5 μM
Nanoporous Pt-Au alloy [63]	Conventional 3-electrode cell: Ref ^1^: Mercury sulfate CE ^2^: Platinum foil	Enhanced electrocatalytic activity towards glucose oxidation and non-enzymatic Simultaneous detection of H_2_O_2_	Highly specialized equipment to produce the Pt-Au alloy and additional preparation for an electrochemical ink Mercury content in the reference electrode which is highly toxic	0.5 μM
Pt surface with nanostructures AuNPs with GOx ^3^ [64]	Conventional 3-electrode cell: Ref ^1^: Ag/AgCl CE ^2^: Platinum foil	Promising applicability for glucose detection in saliva Simultaneous detection of H_2_O_2_	Highly specialized process which use chromium for AuNPs pattering on Pt Uses enzyme and thiol reactants (harmful) for GOx selective immobilization in AuNPs	3.4 nM
Au-Pt bimetallic nanoparticles on SWCNT ^4^ [65]	Conventional 3-electrode cell The Ref and CE were not reported	In-situ synthesis of Au-Pt nanoparticles on working electrode Non-enzymatic sensor Simultaneous detection of H_2_O_2_	Use of a catalytic ink based on highly toxic metals such as cobalt (II) molybdenum (II) Highly specialized equipment for synthesis of SWCNT Use of gold working electrode, which is expensive	50 μM
PTFE ^5^/GOx/PANI ^6^/MWCNT ^7^ on glassy carbon electrode [66]	Conventional 3-electrode cell: Ref ^1^: Ag/AgCl CE ^2^: Platinum foil	High biocompatibility, selectivity, reproducibility, and stability Application for diabetes management: human serum	Uses enzyme, previous synthesis of various components used for the working electrode and sealant needed to maintain the layer-by-layer structure	0.19 μM
Ternary layer of ITO/PbS/SiO_2_/AuNPs [67]	Conventional 3-electrode cell: Ref ^1^: Calomel CE ^2^: Platinum wire	Photoelectrochemical detection of glucose	Use of toxic reactants as lead (II) used for Pbs QD Mercury content in the reference electrode which makes it toxic Use of SiO_2_-NPs modified with thiols (harmful) Previous synthesis of various components used for the working electrode	0.46 μM
G-AuNPs (present work)	CNT-SPE	It is a miniaturized system that only requires 50 μL of sample The system does not require mercury electrodes (calomel) Synthesis of AuNPs is simple and environmentally friendly No enzymes are used, and the modification is very simple	Its detection limit can be an area of opportunity	50 μM

^1^ Reference electrode ^2^ Counter electrode ^3^ Glucose oxidase ^4^ Single-Wall Carbon Nanotubes. ^5^ Poly(tetrafluoroethylene) ^6^ Poly(aniline) ^7^ Multi-Wall Carbon Nanotubes.
